# Sound Range AE as a Tool for Diagnostics of Large Technical and Natural Objects

**DOI:** 10.3390/s23031269

**Published:** 2023-01-22

**Authors:** Yuri Marapulets, Alexandra Solodchuk, Olga Lukovenkova, Mikhail Mishchenko, Albert Shcherbina

**Affiliations:** Laboratory of Acoustic Research, Institute of Cosmophysical Research and Radio Wave Propagation FEB RAS, Kamchatka Region, Elizovskiy District, Mirnaya Str. 7., 684034 Paratunka, Russia

**Keywords:** sound range acoustic emission, combined hydroacoustic receiver, accelerometer, pulse detection, direction detection, adaptive matching pursuit, 43.58.+z, 43.40.Le

## Abstract

Application of acoustic emission of the sound frequency range is under consideration. This range is of current interest for the diagnostics of the stability of mountain slopes, glaciers, ice covers, large technical constructions (bridges, dams, etc.) as well as for the detection of rock deformation anomalies preceding earthquakes. Acoustic sensors, which can be used to record and to determine the directivity of acoustic emission of the sound frequency range, are under consideration. The structure of the system for acoustic emission recording, processing and analysis is described. This system makes it possible to determine the direction to the acoustic emission source using one multi-component sensor. We also consider the algorithms for detection of acoustic emission pulses in a noisy background, and for the analysis of their structure using the Adaptive Matching Pursuit algorithm. A method for the detection of the direction to an acoustic emission signal source based on multi-component sensors is described. The results of application of sound range acoustic emission for the detection of the intensification of rock deformations, associated with earthquake preparation and development in the seismically active region of Kamchatka peninsula, are presented.

## 1. Introduction

Acoustic emission (AE) in solid bodies is elastic oscillations occurring as the result of local dynamic reconstruction of their structure. The main sources of AE are the formation and extension of fractures in brittle solids as well as dislocations in the bodies having more plastic properties. Characteristics of radiated oscillations are directly associated with the peculiarities of the solid body deformation process that determines the interest in the emission investigations to develop the methods of acoustic emission diagnostics. Acoustic emission is applied for different diagnostic problems in seismology, industry (object non-destructive control) and in geophysics. In the wide range of dislocation scales and the corresponding wave lengths of radiated oscillations, we can distinguish three emission frequency ranges, the investigation of which differs both in problems and in observation means. The infrasound frequency range (fractions–units of hertz) is applied to record earthquakes and to estimate their characteristics, and to monitor nuclear tests in seismic measurements [1,2,3]. The ultrasound frequency range from 20–30 kHz to the first megahertz is used in the industry for the early detection of fractures, material creep testing, detection of hidden defects in different types of constructions including the elements of rockets, airplanes, bridges and others as well as in geophysics during the laboratory deformation of rock samples to research fracture formation mechanisms [4,5,6].

The sound range (20 Hz–20 kHz) takes the intermediate position and plays an important role in the interaction of micro and macro dislocations, which is why AE at these frequencies is of special interest when testing large technical and natural objects such as bridges, dams, snow covers, glaciers and mountain slopes. There are cases when this AE range was used to detect rock deformation anomalies preceding earthquakes [7,8].

In the paper, we describe the peculiarities of recording, processing and analysis of AE in the sound range and the results of its application to diagnose the stress–strain state of rocks in the seismically active region of Kamchatka peninsula.

## 2. Recording System for Sound Range Acoustic Emission

The recording system requires the sensors capable of recording AE in the range from the tens of hertz to the tens of kilohertz. In order to determine the direction to the emission source, it is reasonable to use multi-component sensors. Combined hydroacoustic receivers, developed on the basis of vector receivers and hydrophones, and modern accelerometers, have such characteristics.

Combined hydroacoustic receivers are installed by the bottom of natural and artificial water bodies [9]. Results of the experimental research in closed inner water bodies and on the ocean shelf demonstrate that signal form distortion, when propagating in a waveguide (water layer—soil near-surface layer) at short distances, is insignificant [8,10]. There are no transverse oscillations in a fluid; thus, the sensors record only the acoustic wave longitudinal component. That further simplifies the determination of the direction to a emission source. Accelerometers are installed on solid constructions, which are rigidly connected with an object under diagnostics.

**Figure 1:**
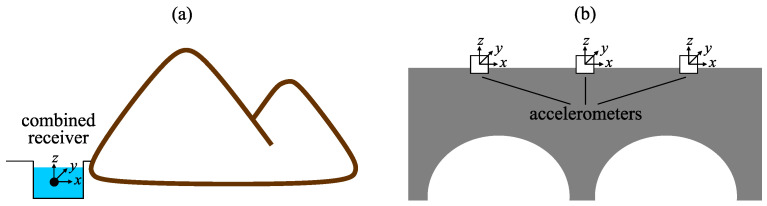
Installation schemes of (**a**) combined receiver and (**b**) accelerometers.

In order to make the diagnostics of the stability of mountain slopes, glaciers, snow covers and to detect rock deformation anomalies, preceding earthquakes, it is more convenient to use combined hydroacoustic receivers. They are installed near an object under the diagnostics in natural and artificial water bodies. In this case, one sensor is enough to make the diagnostics of an object state and to determine the direction to the defect occurring in its structure (Figure 1a). In order to examine large technical constructions, it is better to use the accelerometers arranged on an object surface. In order to determine accurately the direction to the structure defect, it is necessary to use a group of spaced accelerometers (Figure 1b). In this case, the accuracy of defect location depends on the quantity of installed accelerometers.

**Figure 2:**
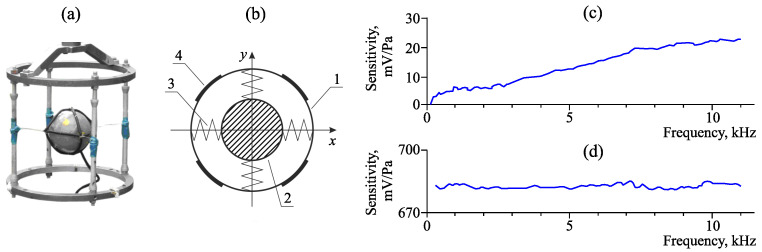
Combined hydroacoustic receiver: (**a**) receiver, (**b**) its block scheme. On the scheme: 1—casing, 2—inertial mass, 3—resilient elements and 4—pressure receiver components. Sensitivity of (**c**) the pressure gradient channels and (**d**) the pressure channel.

Figure 2 illustrates a combined hydroacoustic receiver, its block scheme and sensitivity of its channels. It includes a hydrophone and a three-axis vector receiver of a co-oscillating type, which are optimized for the operation in the sound frequency range with the upper limit of 11 kHz. Inertial mass placed inside its casing is used in the constructions of such devices. An acoustic wave, affecting the casing, makes it move. Due to the inertia, the massive body stands still. If we measure the casing motion velocity vector relatively the inertial mass or the interface forces, it is possible to find the characteristics of the wave affecting the receiver. As a rule, four signals are formed at the output of such type of receivers, they are: acoustic pressure P(t) and three mutually orthogonal components of pressure gradient $P_{x}\left(t\right)$, $P_{y}\left(t\right)$, $P_{z}\left(t\right)$, which are the vector projections of the pressure gradient on the corresponding coordinate axes. Based on these four signals, we can determine the vectors of oscillating speed, shift and acoustic emission power density. The receiver casing is a leakproof spherical body hung on elastic guy lines inside a special frame, which is installed by the bottom of a water body and provides constant orientation in space. To normalize signal levels, the receiver has a multi-channel matching off-casing amplifier.

**Figure 3:**
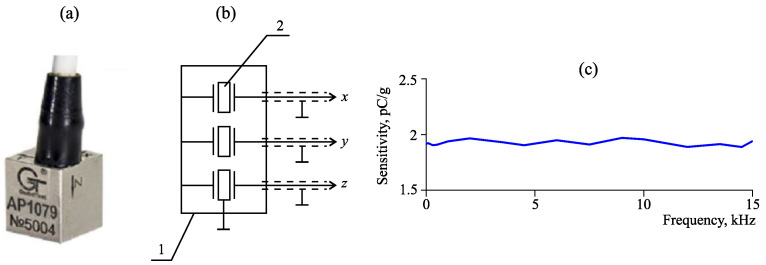
Accelerometer of the model AP1079: (**a**) accelerometer, (**b**) its block scheme and (**c**) sensitivity of channels. On the scheme: 1—casing; 2—inertial mass.

Figure 3 shows an accelerometer of the model AP1079. It is a three-component vibration sensor, which provides acoustic signal transformation into an electric charge proportional to the oscillatory acceleration in the frequency range of 0.5 Hz–15 kHz. Three signals are generated at the output. They correspond to the orthogonal components of oscillatory acceleration vector of an object under control.

**Figure 4:**
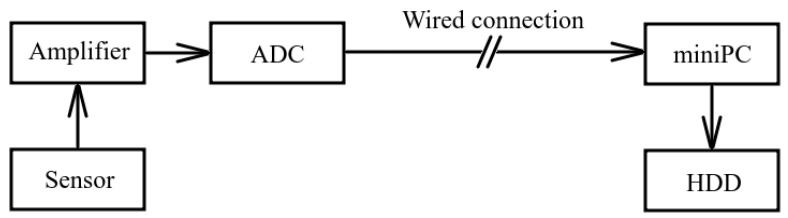
Block scheme of the acoustic receiving system with wired connection.

In order to record and to collect acoustic data, a system was developed. Its block scheme is illustrated in Figure 4. One of the suggested sensors was used as a recording unit. It is installed at the point of acoustic signal reception. When an acoustic signal have been amplified and digitized, it is sent to a minicomputer via a data transmission line and then is stored on a PC hard disk. Signal can be transmitted via a cable line. It is possible to transmit data at the distance up to 200 m by a wired connection.

**Figure 5:**
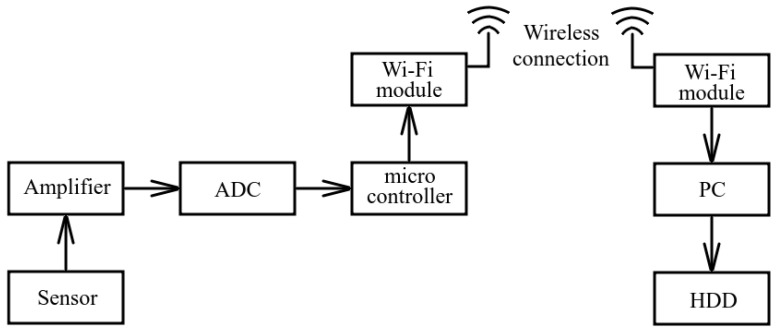
Block scheme of the acoustic receiving system with data transmission channel based on Wi-Fi modules.

When there is no possibility to make a wired connection channel, data can be transmitted via a wireless Wi-Fi channel. In that case, the receiving system is modified by adding Wi-Fi modules (Figure 5). Such a scheme has recorded data transition limits within a line of sight at the distance up to 1–2 kilometers.

**Figure 6:**
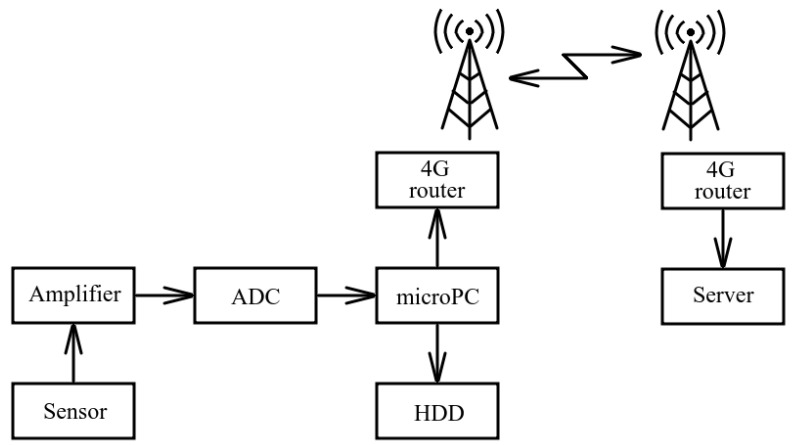
Block scheme of the spaced system for acoustic data recording.

In order to organize data collection at widely-spaced sites for AE signal observations, we proposed to deploy a spaced system (Figure 6). It includes an autonomous branch, located at a remote observation site, and a stationary branch [11].

An autonomous branch of the system receives acoustic signals, digitizes them, records on a hard disk connected to a microcomputer and transmits the data to the system stationary branch. The stationary branch receives the data and stores them on a server for further processing. Spaced branches of the system can be united into a single network by a communication channel based on mobile internet technology.

This scheme of the receiving system also has limits in data transition. That is associated with the fact that remote observation sites should be located within the zone of the stable reception of a mobile connection network of 4G generation and higher.

## 3. Acoustic Emission Signal Processing

To develop methods for the processing and analysis of sound range AE signals, we investigated the characteristics of the signals occurring during rock deformation [9,12]. It was stated that a typical AE signal is composed of a sequence of relaxation pulses of different amplitude and duration with shock excitation and the filling frequency from hundreds of hertz to ten kilohertz. Thus, to investigate the deformation process, the important characteristics are the AE pulse amplitude, repetition rate, form and frequency filling. The amplitude is determined by the intensity of source deformation intensity and the distance to it. Pulse repetition rate is also determined by deformation and may change within wide limits from single signals on the time interval of several seconds during a “background” period to the tens and even hundreds per a second during deformation anomalies. The most informative part of a pulse is the front and the decay beginning, which allow one to determine the direction to a emission source, and the filling frequencies contain information on its size and dynamics [12]. Thus, the important task of AE signal processing is the detection of pulses in a noisy background.

### 3.1. Pulse Detection

In a general case, a digitized signal of acoustic emission can be described as a sum of noise ε(n) and some signal s(n), the analytical expression of which is unknown,

(1) x(n)=s(n)+ε(n),

where ε(n) describes nonstationary background noise, s(n) is the “useful” signal, which represents a sequence of pulses of different form, amplitude and frequency, and *n* is the normalized discrete time.

Then, x(n) can be represented as

(2) $$x\left(n\right)=\sum_{i=0}^{K-1}g_{i}\left(n-m_{i}\right)+\varepsilon \left(n\right),$$

where *K* is the number of pulses, $g_{i}\left(n\right)$ is the function describing the *i*-th pulse, and $m_{i}$ is the start time of the *i*-th pulse.

To detect pulses from the signals under investigation, we propose a threshold scheme [13]. The pulse beginning and end are determined by comparing signal x(n) amplitude with the threshold value *S*. As long as the threshold value should depend on the background noise level, its equation takes into account the expectation and the root-mean-square deviation of signal noisy fragments. The current threshold value is estimated in noncrossing windows of a fixed width of *N* samples

(3) $$S_{k}=\mu_{k-1}+A\sigma_{k-1},$$

where *k* is the window number, $S_{k}$ is the threshold value calculated in *k*-th window, $\mu_{k-1}$ is the expectation of the amplitudes of previous *N* samples, $\sigma_{k-1}$ is the root-mean-square deviation of the amplitudes of the previous *N* samples, and *A* is the parameter determined experimentally.

**Figure 7:**
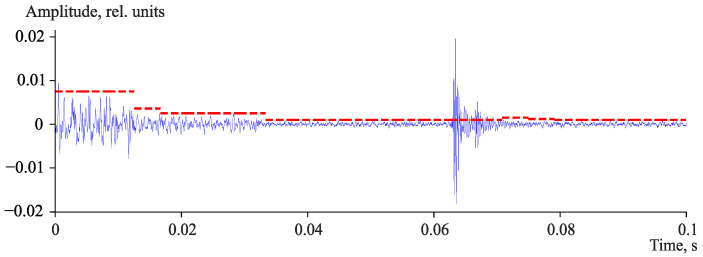
Example of threshold value adaptation to background noise level. Dashed red line is the threshold.

In order to provide the dependence of the threshold value only on noise level, the detected pulse samples are removed from the sequence of *N* samples. The current threshold value holds until the collected data are enough to estimate a new value. It was empirically found that *N* value lies in the range from 200 to 400 samples and the parameter *A* value is in the range from 3.1 to 3.8 for the signals under investigation. An example of threshold value adaptation to noise level is illustrated in Figure 7.

**Figure 8:**
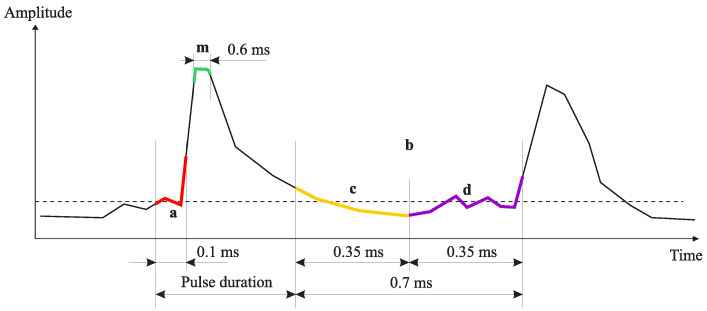
Example of threshold value adaptation to background noise level.

Pulse detection algorithm is the following. Pulse front is fixed when signal average amplitude, estimated in the window of the width of 0.1 ms, exceeds the threshold *S* (Figure 8, segment **a**). Signal amplitude averaging removes false responses, which occur as the result of the recording of non-systematic short emissions determined by noises, as a rule.

Pulse end is stated when signal amplitude becomes less than the operating threshold. Quite low frequencies can be observed in pulse droop, which is why its amplitude will be less than the threshold at a significant time interval. In order to avoid the early detection of pulse end, we carry out the search by analyzing the signal amplitude maximum in the sliding time window with the duration of 0.7 ms (Figure 8, segment **b**).

**Figure 9:**
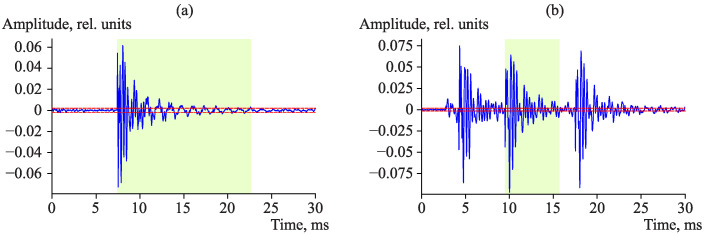
Examples of pulse detection from AE signal: (**a**) single pulse; (**b**) pulse of a sequence. Solid blue line shows the signal; dashed red line is the threshold. Green area shows the boundaries of the detected pulse.

The situation is typical for AE signals when pulses are close to each other, thus, simultaneously with the search for the pulse ending, we look for the beginning of the next pulse by comparing average amplitudes of a signal in the current and in the next windows with the duration of 0.35 ms (Figure 8, segments **c**,**d**). If we detect an increase in the amplitude average value by more than 1.5 time in the next window, we make a decision on the end of the current pulse and the beginning of the next one. In this case, the algorithm begins to look for the end of the next pulse at once. Figure 9 shows the examples of the detection of a single pulse and a pulse of a sequence. The signal amplitude, illustrated in Figure 9b, does not drop below the threshold but the difference in signal average amplitudes at about 15 ms is fairly interpreted by the algorithm as the end of the current pulse and the beginning of the next one.

Two types of errors are possible when detecting pulses, they are: pulse loss (I-st type error) or false response of the algorithm (II-nd type error). Table 1 shows the test results of the threshold scheme on a model signal containing 100 pulses at different noise levels. Tests demonstrated that the most critical error of the false detection is almost excluded.

The presented algorithms for pulse detection can be used with any AE sensors including combined hydroacoustic receivers and accelerometers.

### 3.2. Time-Frequency Processing

Further investigation of the detected pulses suggests their time-frequency processing. Additional difficulties arise at this stage. They are associated with a wide variety of pulse waveforms, short duration and strong noisiness by natural and industrial sources [14]. Thus, the application of time-frequency analysis methods used to solve such problems in allied science fields (Short Time Fourier transform [15], wavelet transform [16], wavelet packets [17] etc.) is of low efficiency [18]. We suggest a new approach to the time-frequency analysis of AE signals. It is based on a sparse approximation method applying the Adaptive Matching Pursuit algorithm [14]. The main idea of sparse approximation is signal representation in the form of a finite linear combination of functions from some large set linearly dependent in the general case. The difference from a simple approximation is that the decomposition includes not all functions but only some of them, i.e., the decomposition coefficient vector contains a large number of zero coefficients.

(4) $$\left\{\begin{array}{c} x\left(t\right)=\sum_{i=0}^{N-1}a_{i}g_{i}\left(t\right)\\ {∥a∥}_{0}\to \min \end{array}\right.,$$

where x(t) is the signal, $g_{i}\left(t\right)$ are the functions, into which the signal is decomposed, $a_{i}$ are the decomposition coefficients, *N* is the number of functions into which the signal x(t) is decomposed and ${∥a∥}_{0}=\#\left\{i:\;a_{i}\neq 0,\;i=0\ldots N-1\right\}$ is the $l_{0}$-pseudonorm equal to the number of vector non-zero elements.

The problem of the search for the exact solution of the system (4) is NP-hard. However, there are methods allowing one to obtain the estimate of the exact solution with some error for polynomial time. One of such methods is the Matching Pursuit method substituting the $l_{0}$-norm minimization by the upper bound. The main disadvantage of the method is its high calculation complexity $O(N^{2}\log N)$. We suggest a modified version of the method, Adaptive Matching Pursuit, which allows us to obtain the solutions of the same accuracy on function sets of smaller sizes [14].

The choice of a function set $g_{i}\left(t\right)$ affects the approximation quality significantly. Thus, the necessary condition is the correspondence of their forms to real signals when selecting the functions. For example, in the seismic survey for the analytical description of seismic-acoustic oscillations, they often use Gaussian pulses

(5) $$g\left(t\right)=\exp\left(-Bt^{2}\right)\cos\left(\omega t\right),\;-\infty <t<+\infty ,$$

where *B* is the parameter affecting pulse envelope attenuation rate, ω is the cycle frequency and Berlage pulses

(6) $$g\left(t\right)=t^{n}\exp\left(-Bt\right)\sin\left(\omega t\right),\;0\leq t<+\infty ,$$

where *B* is the parameter affecting pulse envelope attenuation rate, ω is the cycle frequency. That is why these functions were used for the sparse approximation of sound range AE signals [19].

**Figure 10:**
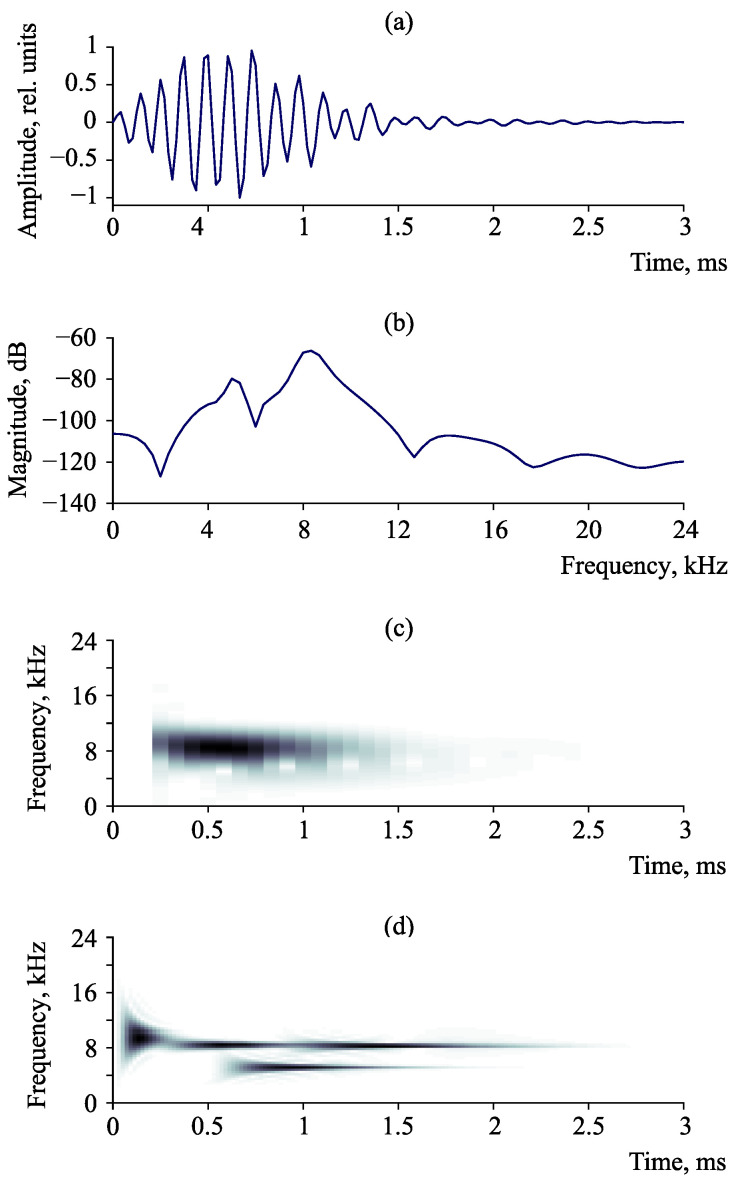
Comparison of different methods of frequency and time-frequency analysis: (**a**) signal, (**b**) estimate of spectral power density by Fast Fourier transform, (**c**) Short Time Fourier transform and (**d**) sparse approximation (Adaptive Matching Pursuit).

Figure 10 shows the comparison of different methods for time-frequency analysis on the example of a model signal. Signal characteristics are the following: sampling rate is 48 kHz, duration is 3 ms, and consists of thee pulses with the frequencies of 5, 8, 8.5 kHz (Figure 10a). The estimate of the spectral power density by Fast Fourier transform allows us to detect only two frequencies out of three (Figure 10b). Thee frequencies merge into one structure on the signal spectrogram (Figure 10c). The application of sparse approximation (Figure 10d) makes it possible to detect all three frequencies in the signal. Further, the sparse approximation allows us to analyze the frequency content of pulses detected in a noisy background. That is necessary in the determination of the defect scale in space under the control as long as pulse frequency is determined by the AE source size [19].

### 3.3. Determination of the Direction to Emission Source

**Figure 11:**
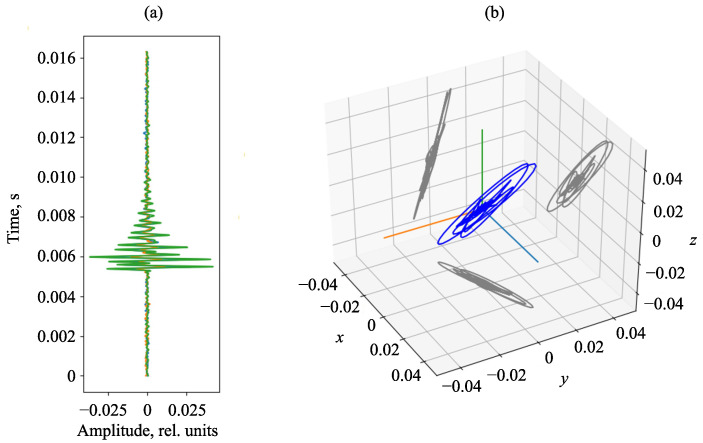
Example of AE pulse: (**a**) pulse; (**b**) hodograph of acoustic pressure gradient represented in three-dimensional coordinates with the projections on axial planes. In the figure, X, Y and Z are coordinate axes.

We consider a hodograph of the acoustic pressure gradient of an AE pulse recorded by the combined hydroacoustic receiver. The hodograph has a clear elliptical form (Figure 11), which can be used to detect the direction to an AE pulse source.

**Figure 12:**
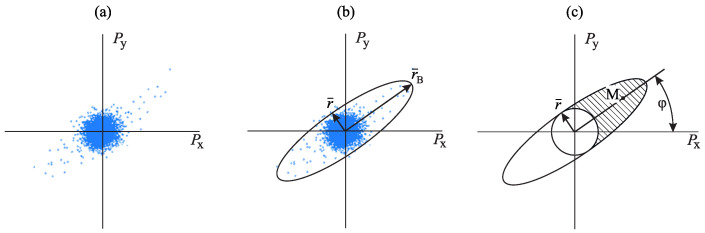
Determination of the direction to a signal source: (**a**) projections of pulse samples on the XY plane, (**b**) mapping of the describing ellipse and determination of noise level and (**c**) elimination of count direction ambiguity, estimation of mass center *M* and determination of azimuth on the signal source.

To solve this problem in the horizontal XY plane, an amplitude method is used [20]. According to it, pulse samples are mapped in Cartesian coordinate system (Figure 12a). In this case, they are grouped in a bounded domain, which can be described by an ellipse (Figure 12b). The ellipse major semi-axis ${\bar{r}}_{B}$ will be directed along the axis of the signal-receiver signal. The minor semi-axis $\bar{r}$ will roughly correspond to noise level (Figure 12b).

After that, direction ambiguity for each sample of a pulse is eliminated by analyzing the phase difference of the signals recorded by vector channels and the acoustic pressure channel [21]. As the result of this operation, the majority of the samples with the amplitudes, exceeding the background value, are grouped in one half of a describing ellipse along its major semi-axis (Figure 12c). Determination of the direction to a signal source is reduced to the calculation of the direction to the mass center *M* of the points from the described ellipse-shaded area (Figure 12c).

(7) $$\tan\varphi =\frac{M_{y}}{M_{x}},$$

where φ is the azimuth, $M_{x},M_{y}$ are projections of mass center *M* on X and Y axes.

Mass of each point *m*, used in the determination of mass center, is not similar and is estimated by the formula

(8) $$m=\frac{r_{M}}{\bar{r}},$$

where $r_{M}$ is the length of the mass center radius-vector, $\bar{r}$ is the minor semi-axis length of the described ellipse.

Introduction of unequal masses for points allows us to give priority to the samples with the highest amplitudes, which carry the main information on the direction to a signal source. In order to realize this method in space, besides the analysis of the pulse projections on the horizontal XY plane, we also consider projections on two mutually orthogonal vertical planes. After that, three-dimensional coordinates of mass center *M* are estimated and the direction to a source is determined.

We should note that this method is applicable when using a combined hydroacoustic receiver. In the case of accelerometers, the ellipse, describing signal hodograph, is also mapped. However, as long as there is no additional channel of acoustic pressure, it is necessary to make a network of several sensors, located at some distance from each other, to eliminate direction ambiguity. The correct direction is determined by finding the intersection area of ellipses main axes from each sensor.

## 4. Application of Sound Range Acoustic Emission in Rock Diagnostics

**Figure 13:**
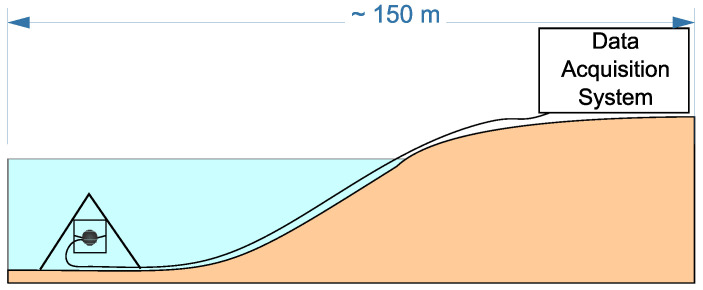
Scheme of the combined receiver installation by the bottom of Mikizha lake [19].

As an example of the application of the sound range AE in natural object diagnostics, let us consider its application in the investigation of the rock stress–strain state before earthquakes. In order to do that, a combined hydroacoustic receiver (Figure 2) was installed by the bottom of Mikizha lake (52.99${}^{\circ }$ N, 158.23${}^{\circ }$ E) in the seismically active region of the Kamchatka peninsula. A scheme of the receiver setup is illustrated in Figure 13. This lake is located in the region of a tectonic fault, and cases of anomalous pre-seismic disturbances of AE have been multiply recorded at this point before [22,23,24]. Acoustic activity Ω(t) (AE pulse recurrence rate per a time unit) and its azimuthal distribution D(φ,t) at different stages of a seismic process were under the analysis. In the course of the experiments, cases of anomalous change in these parameters before and after earthquakes were detected and compared with seismically calm periods. Examples of the anomalies are describe below.

**Figure 14:**
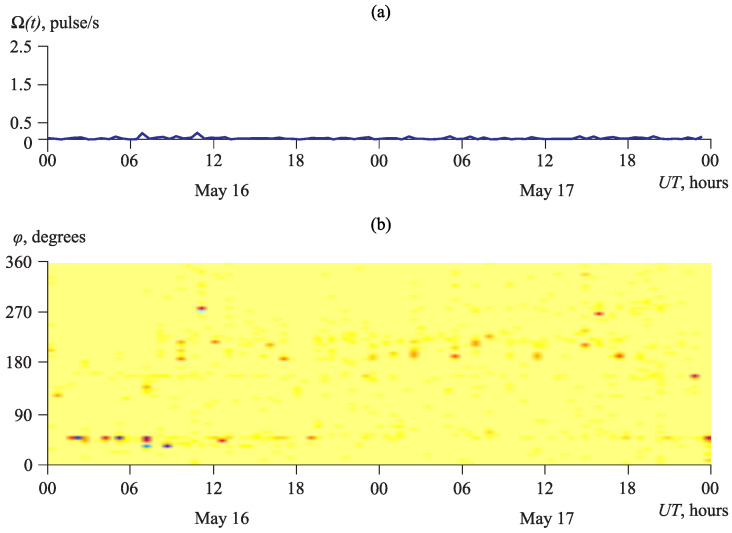
Seismically calm period on 16 and 17 May 2009: (**a**) acoustic activity Ω(t) and (**b**) its azimuthal distribution D(φ,t). The color shows the number of detected pulses ascending from white to cyan.

During seismically calm periods, AE pulse sources are located quite uniformly with respect to directions. Figure 14 illustrates the acoustic activity Ω(t), averaged one time per a second, and its azimuthal distribution D(φ,t) on 16 and 17 May 2009.

On these calm days, the average level of acoustic activity did not exceed the value of 0.01 pulse/s (Figure 14a). The increase in the pulse number in separate directions was not almost observed on the graph of the azimuthal distribution (Figure 14b).

**Figure 15:**
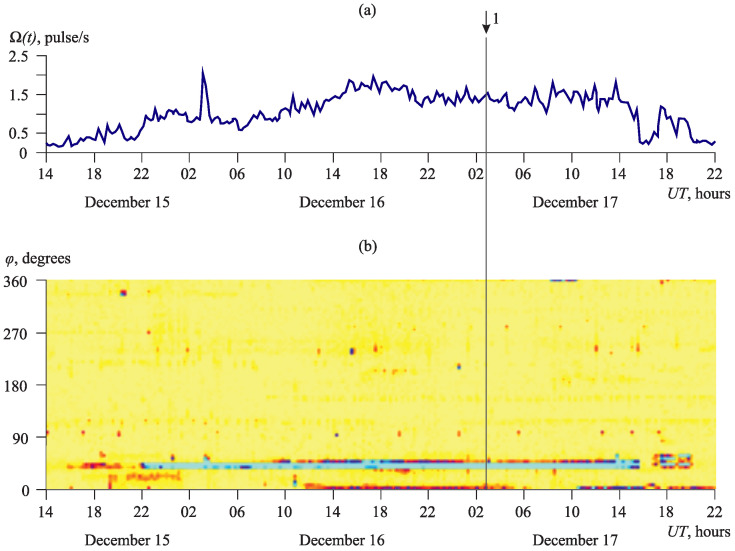
Period before and after the earthquake with $M_{l}=4.4$ occurred on 17 December 2012: (**a**) acoustic activity Ω(t) and (**b**) its azimuthal distribution D(φ,t). The color shows the number of detected pulses ascending from white to cyan.

**Figure 16:**
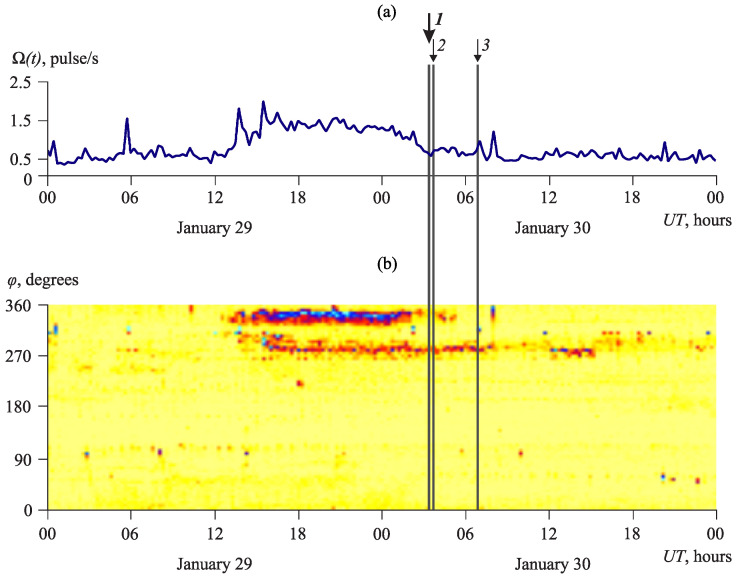
Period before and after the earthquake with $M_{l}=7.1$ (1) and its aftershocks (2, 3) occurred on 30 January 2016: (**a**) acoustic activity Ω(t), (**b**) its azimuthal distribution D(φ,t). The color shows the number of detected pulses ascending from white to cyan.

During the intensification of the deformation processes, including those caused by earthquakes, the increase in acoustic activity is observed. Diagrams of its azimuthal distribution change significantly. Maximums in separate directions are clearly expressed (Figure 15 and Figure 16). Figure 15 shows the anomaly recorded 30 h before the earthquake with local magnitude $M_{l}=4.4$, which occurred at 03:10 UT on 17 December 2012 [25]. Epicenter coordinates are 51.88${}^{\circ }$ N, 159.12${}^{\circ }$ E, the depth is 64 km, and the epicentral distance is 138 km (1 on the graph). The anomaly was over 17 h after the earthquake (the anomaly duration was 47 h). A sharp increase in the AE pulse recurrence rate is observed on the graph of acoustic activity (Figure 15a). Activation of the emission from the directions in the range φ from 40${}^{\circ }$ to 50${}^{\circ }$ corresponds to this increase (Figure 15b).

Figure 16 shows AE directivity anomalies associated with the earthquake with local magnitude $M_{l}=7.1$ [25]. This earthquake occurred at 03:25 UT on 30 January 2016. The epicenter coordinates are 53.85${}^{\circ }$ N, 159.04${}^{\circ }$ E, the depth is 178 km, the epicentral distance is 110 km (1 on the graph). Two strong aftershocks of this earthquake were also recorded [25]. The first one occurred with $M_{l}=5.1$ at 03:42 UT on 30 January 2016. The epicenter coordinates are 53.76${}^{\circ }$ N, 159.08${}^{\circ }$ E, the depth is 184 km, and the epicentral distance is 101 km (2 on the graph). The second one occurred with $M_{l}=4.5$ at 06:53 UT on 30 January 2016. The epicenter coordinates are 53.88${}^{\circ }$ N, 159.22${}^{\circ }$ E, the depth is 175 km, and the epicentral distance is 117 km (3 on the graph).

Anomalies in the direction range φ from 330${}^{\circ }$ to 350${}^{\circ }$ (Figure 16b), during which a sharp increase in acoustic activity level was observed (Figure 16a), began 15 h before the earthquake with $M_{l}=7.1$ and were over at about 30 min before it. Almost simultaneously with it, a somewhat less increase in the activity was recorded in the azimuth of 280${}^{\circ }$ (Figure 16b). It lasted after the earthquake and is likely to be associated with the presence of aftershocks.

As a result of the analysis of a long-term series of AE observations, the authors developed a method for detecting anomalies of acoustic activity associated with earthquakes [26]. The method consists in estimating the values of acoustic activity and its azimuthal distribution in calm weather conditions (slightly changing atmospheric pressure, absence of precipitation and wind more than 6 m/s). If the azimuthal distribution of acoustic activity has maxima in one or several directions and at the same time the repetition rate of acoustic pulses exceeds the background level by 2.5 or more times for at least 6 h, then an earthquake will occur in the next 10–30 h with a probability of more than 70% [26].

The presented examples demonstrate that the suggested solutions on the recording and processing of the data of AE in the sound frequency range were effective enough for the diagnostics of the rock stress–strain state before earthquakes.

## 5. Conclusions

AE of the sound frequency range is suggested to be used for the diagnostics of the stability of mountain slopes, glaciers, snow covers, large technical constructions (bridges, dams, etc.) and for the detection of rock deformation anomalies preceding earthquakes. This paper considers the peculiarities of the construction of a system for sound range AE signal recording and processing. Acoustic sensors, which can be used for the recording and determination of the direction to AE sources, are described. Two types of sensors were suggested, they are: combined hydroacoustic receivers and accelerometers. It was demonstrated that when using a combined hydroacoustic receiver, one sensor is enough to determine the direction to a emission source. In case of using accelerometers to eliminate the direction ambiguity, it is necessary to install several sensors located at some distance from each other. To make the diagnostics of the stability of mountain slopes, glaciers, snow covers and to detect rock deformation anomalies, preceding earthquakes, it is more convenient to apply combined hydroacoustic receivers. For the diagnostics of large technical constructions, it is better to use accelerometers mounted on an object surface.

The algorithms for the detection of AE pulses in a noisy background, for the analysis of the time-frequency structure of pulses and for the determination of the direction to the AE source, were proposed. Examples of the application of the sound range AE for the detection of the activation of rock deformation, associated with earthquake preparation in the seismically active region of Kamchatka peninsula, were presented. We think that the suggested tools can be helpful when developing systems for the diagnostics of the stability of different large technical constructions and natural objects.

## Figures and Tables

**Table 1:** Test results for the pulse detection scheme.

Signal-to-Noise Ratio, dB	Number of Detected Pulses	I-st Type Errors	II-nd Type Errors	Number of Correctly Detected Pulses
20	100	0	0	100
15	97	3	0	97
10	85	15	0	85
5	65	35	0	65
0	36	64	0	36
−5	0	100	0	0

## Data Availability

The data on earthquakes considered in the article are available at the Unified Information System of Seismological Data of the Kamchatka Branch of Geophysical Service Russian Academy of Science at http://sdis.emsd.ru/info/earthquakes/catalogue.php (accessed on 15 November 2022).
